# Irritability in Bipolar Depression: Re‐Examining a Neglected Dimension

**DOI:** 10.1111/bdi.70178

**Published:** 2026-09-01

**Authors:** Francesco Attanasio, Caterina Fumagalli, Rocco Miazzi, Valentina Fazio, Cristina Colombo

**Affiliations:** ^1^ Department of Clinical Neurosciences Vita‐Salute San Raffaele University Milan Italy; ^2^ Department of Human Neuroscience Sapienza University of Rome Rome Italy; ^3^ Mood Disorder Unit, Department of Clinical Neurosciences IRCCS San Raffaele Scientific Institute Milan Italy

“It feels like my nerves are constantly on edge”, “It's a continuous tension that makes me snap over nothing”, “Even the people I love irritate me, I can't control it.” These are common expressions voiced by patients with bipolar disorder (BD), reflecting a pervasive yet poorly understood experience: irritability.

## 1 A Conceptual Challenge

Despite its prevalence, irritability lacks a clear and consistent definition and is often conflated with related constructs such as *anger*, *aggression*, or *frustration* [1, 2]. This conceptual ambiguity is also mirrored in diagnostic nosology. The Diagnostic and Statistical Manual of Mental Disorders, Fifth Edition, Text Revision, refers to “*irritable mood*” and “*irritable behavior*” across several diagnostic categories but fails to provide a consistent or operational definition. Such inconsistency not only reflects broader theoretical uncertainty but also complicates how irritability is recognized and assessed in both clinical and research settings.

These challenges gain particular relevance in the context of BD. More than a century ago, Kraepelin (1921) described irritability as “a vulnerability to manic‐depressive illness”, conceptualizing it as a form of inner tension bridging the manic and depressive poles. His early insight anticipated contemporary perspectives [3, 4] that frame irritability as a transpolar symptom within the bipolar spectrum, one that cuts across affective phases and modulates clinical expression, thereby challenging the traditional view of depression and mania as strictly opposite poles.

Despite this long‐standing recognition, research on irritability has remained unbalanced. Most studies have focused on its manifestation during manic and mixed episodes, where it is both part of the diagnostic criteria and a salient clinical feature [3, 4]. By contrast, its manifestation in bipolar depression, which lacks explicit diagnostic recognition, has been comparatively neglected and remains systematically understudied, highlighting a significant gap in the literature. Nevertheless, existing empirical evidence indicates that the presence of irritability is associated with poorer quality of life, greater psychosocial dysfunction, higher levels of aggression and suicidality, and reduced treatment adherence [4].

Irritability has also been proposed as a potential discriminant between unipolar and bipolar depression. Mesbah et al. [5] reported that elevated irritability and anger may serve as early clinical indicators of BD. Conceptualizing irritability as a transpolar and transdiagnostic dimension could therefore improve early differential diagnosis between unipolar and bipolar depression, two conditions that require distinct management strategies and entail different clinical implications.

## 2 A Challenge for Measurement and Research

This clinical recognition remains constrained by how irritability is currently assessed. In adult mood disorder populations, irritability has often been evaluated only through single items within broader scales, such as the Young Mania Rating Scale, the Generalized Anxiety Disorder Scale, the Hamilton Depression Rating Scale, or the Inventory of Depressive Symptomatology—Self‐Report. While convenient, these one‐item approaches are overly reductive and fail to capture the multidimensional nature of the symptom. Moreover, they do not provide a clear description or shared definition of what “irritability” entails, preventing a consistent evaluation of this construct across clinical settings. Occasionally, irritability has been explored using structured interviews or personality questionnaires, which tend to capture enduring traits rather than state‐dependent manifestations. Several multi‐item self‐report scales have been developed or adapted in recent years to more specifically assess irritability in mood disorder populations, including the Affective Reactivity Index, the Brief Irritability Test, the Brief Self‐report Irritability Scale, and the Concise Associated Symptom Tracking—Irritability Subscale. These instruments demonstrate promising psychometric properties; however, their application remains limited, validations are often restricted to specific contexts and languages, and several items risk capturing overlapping constructs such as impulsivity or anxiety. Despite the evident need, reflected by the proliferation of instruments attempting to capture this construct, no consensus has been reached on a standardized measure. More importantly, none has been specifically validated for bipolar depression, leaving a critical gap in both conceptual understanding and clinical assessment.

## 3 A Challenge for the Future?

Most mental health professionals working with BD have likely found themselves writing the word *“irritability”* in their clinical notes, acknowledging its clinical relevance, yet lacking a consistent understanding of its definition, how it should be addressed, or what its presence truly represents in the patient's life and in the longitudinal course of the illness.

These uncertainties can be viewed not merely as obstacles, but as crucial guides for future research. Future studies should aim to establish a clear and operational definition of irritability, distinct from overlapping constructs such as anger or aggression [1, 2]. Equally important is the development and cross‐cultural validation of specific instruments for the assessment of irritability, validated within distinct clinical populations such as BD, where the construct may display unique phenomenological expressions compared with other psychiatric conditions.

The fact that irritability has not been specifically investigated during bipolar depression represents both a challenge and an opportunity for the future optimization of phase‐specific therapeutic interventions. Bipolar depression is still too often conceptualized, and consequently treated, as if it were unipolar depression, despite being a distinct clinical entity that shares only the superficial label of “depression.” It therefore carries unique features and requires tailored approaches that remain, at least in part, to be discovered.

A refined understanding of this transdiagnostic and “transpolar” dimension could enhance diagnostic precision, particularly in differentiating unipolar from bipolar depression during the early stages of illness [5], while reducing trial‐and‐error approaches and the burden of long‐term maintenance therapies.

In conclusion, adequately addressing irritability in BD is therefore not merely an academic exercise but a clinical responsibility toward our patients, with the potential to improve both the understanding and the management of bipolar depression, ultimately leading to better quality of life and long‐term outcomes.

## Data Availability

Data sharing not applicable to this article as no datasets were generated or analysed during the current study.

## References

[1] B. Saatchi , E. F. Olshansky , and M. A. Fortier , “Irritability: A Concept Analysis,” International Journal of Mental Health Nursing 32, no. 5 (2023): 1193–1210, 10.1111/inm.13140. 36929104

[2] M. J. Toohey and R. DiGiuseppe , “Defining and Measuring Irritability: Construct Clarification and Differentiation,” Clinical Psychology Review 53 (2017): 93–108, 10.1016/j.cpr.2017.01.009. 28284170

[3] E. Bell , P. Boyce , R. J. Porter , R. A. Bryant , and G. S. Malhi , “Could Irritability Be the Key to Unlocking the Enigma of Mixed States?,” Bipolar Disorders 22, no. 8 (2020): 781–784, 10.1111/bdi.13029. 33113262

[4] L. Berk , K. T. Hallam , K. Venugopal , et al., “Impact of Irritability: A 2‐Year Observational Study of Outpatients With Bipolar I or Schizoaffective Disorder,” Bipolar Disorders 19, no. 3 (2017): 184–197, 10.1111/bdi.12486. 28470892

[5] R. Mesbah , N. de Bles , N. Rius‐Ottenheim , et al., “Anger and Cluster B Personality Traits and the Conversion From Unipolar Depression to Bipolar Disorder,” Depression and Anxiety 38, no. 6 (2021): 671–681, 10.1002/da.23137. 33503287 PMC8248435

